# Nanomaterials as Analytical Tools for Genosensors

**DOI:** 10.3390/s100100963

**Published:** 2010-01-26

**Authors:** Khalid M. Abu-Salah, Salman A. Alrokyan, Muhammad Naziruddin Khan, Anees Ahmad Ansari

**Affiliations:** King Abdullah Institute for Nanotechnology, King Saud University, Riyadh-11451, P.O Box-2454, Saudi Arabia; E-Mails: abusalah@ksu.edu.sa (K.M.A.-S.); salrokyan@ksu.edu.sa (S.A.A.); mnkhan_phy@yahoo.com (M.N.K.)

**Keywords:** nanomaterials, oligonucleotides, ZnO, carbon nanotubes, conducting polymers

## Abstract

Nanomaterials are being increasingly used for the development of electrochemical DNA biosensors, due to the unique electrocatalytic properties found in nanoscale materials. They offer excellent prospects for interfacing biological recognition events with electronic signal transduction and for designing a new generation of bioelectronic devices exhibiting novel functions. In particular, nanomaterials such as noble metal nanoparticles (Au, Pt), carbon nanotubes (CNTs), magnetic nanoparticles, quantum dots and metal oxide nanoparticles have been actively investigated for their applications in DNA biosensors, which have become a new interdisciplinary frontier between biological detection and material science. In this article, we address some of the main advances in this field over the past few years, discussing the issues and challenges with the aim of stimulating a broader interest in developing nanomaterial-based biosensors and improving their applications in disease diagnosis and food safety examination.

## 1. Introduction

Nucleic acid biosensors have become increasingly prominent in the literature because of the opportunities they offer for better diagnosis, prevention and treatment of many human diseases [1–5]. The detection of genetic disorders is clearly of upmost importance for preventative health care [1,2]. Preventing and treating human diseases is essential for designing reliable efficient and inexpensive tools for determining the genomic sequences, which have broad potential applications including gene expression monitoring, pharmacogenomic research and drug discovery, clinical diagnostics, viral and bacterial identification, detection of bio-warfare and bioterrorism agents, and forensic and genetic identification, *etc*. [4]. To exploit these opportunities, a variety of assays for DNA detection have been developed [3–9]. Molecular diagnostics based on the analysis of genomic sequences have offered a highly sensitive and quantitative method for the detection of infectious disease pathogens and genetic variations. Conventional methods for the analysis of specific gene sequences are based on either direct sequencing or DNA hybridization. Because of its simplicity, the DNA hybridization technique is more commonly used in the diagnostic laboratory than the direct sequencing method. In DNA hybridization, the target gene sequence is identified by a DNA probe that can form a double-stranded hybrid with its complementary nucleic acid with high efficiency and extremely high nucleic acid with high efficiency and extremely high specificity in the presence of a mixture of many different, non-complementary nucleic acids. DNA probes (sometimes called nucleic acid probes or gene probes) are single-stranded oligonucleotides labeled with either radioactive or non-radioactive materials to provide detectable signals for DNA hybridization [10].

Among the conventional DNA detection techniques, electrochemical biosensors represent a leading approach for fast and sensitive determined of the genetic disorder [7]. Due to their high specificity, speed, portability, and low cost, electrochemical biosensors offer exciting opportunities for numerous decentralized clinical applications, ranging from ‘alternative-site’ testing emergency-room screening, bedside monitoring, or home self testing [6–8]. Electrochemical devices have traditionally received the major share of the attention in biosensor development. Such devices produce a simple, inexpensive and yet accurate and sensitive platform for patient diagnosis [4,5].

The name electrochemical biosensor is applied to a molecular sensing device which intimately couples a biological recognition element to an electrode transducer. The purpose of the electrochemical transducer is to convert the biological recognition event into a useful electrical signal. To continue these advances, for utilization of these opportunities, and to move DNA diagnostics out of the central laboratory, future devices must link high performance (particularly high sensitivity and selectivity), with high speed, miniaturization, and low cost [6–9]. The realization of such powerful devices requires innovative efforts in the development of new material design and novel fabrication processes. In this respect, various conventional macromolecular material matrices have been proposed for the development of electrochemical DNA biosensing devices. Such electrochemical biosensing devices have some safety problems like as poor sensitivity, selectivity and low stability associated with the radioisotopic, fluorescent, and enzyme labels.

In particular, nanostructured materials are opening new horizons for the application of electrochemical DNA biosensors. The applications of nanostructured materials in electrochemical biosensors have been reviewed recently [11–20]. They proved the nanostructured materials are extremely useful in the fabrication of electrochemical DNA biosensing devices. There are many reports are available in literature on direct electrochemistry of redox active probe single strand DNA (ssDNA) immobilized onto nanoparticle-modified electrodes [12,18–20]. These nanostructured modified electrodes not only improve the catalytic activity of the transducer but also promote the enzymatic reaction on the electrode surface. The enhanced electrochemistry is due to the ability of the small nanoparticles to reduce the distance between the redox site of a protein and the electrode, since the rate of electron transfer is inversely dependent on the exponential distance between them. A range of nanostructured materials including nanotubes, nanobelt, nanofibers, nanorods, nanocomb and nanowires, prepared from metals, semiconductor, carbon or polymeric species, have been widely investigated for their ability to enhance the response of biosensors [21–24]. Nanoparticles can be used in a variety of ways, such as modification of electrode surfaces, or to modify biological receptor molecules such as enzymes, antibodies or oligonucleotides (ODNs). Some successes of nanostructured materials have been ascribed to their ability to improve the features of bioassays, allowing miniaturization and speed, reducing reagent and sample consumption, and facilitating the performance of heterogeneous formats [12]. The use of nanostructured materials therefore allows miniaturization of biosensors, development of microfluidic systems and increase in the sensitivity of bioassays.

This review is focused on the development and validation of portable electrochemical DNA biosensors that incorporate nanomaterials as either a signal transducer or as an electroactive species for direct detection of analyte. Given the sensitivity, flexibility, and miniaturization capabilities, these sensors have the potential to become the next generation of field-deployable analytical instruments. Our intent is to provide a general overview on nanostructured materials based electrochemical DNA biosensors and their success in detection and quantifications of different forms of DNA. In this review, we have highlighted the recent developments of nanotechnology-based electrochemical DNA biosensors for detection and quantification of biomarkers of exposure or disease and discuss future considerations and opportunities for advancing the use of electrochemical sensors for dosimetric studies.

## 2. Electroanalytical Properties of Nanoscale Materials in Biosensing

Electroanalytical properties of nanoscale materials are very important for biosensing applications, as well as for understanding the unique one-dimensional carrier transport mechanism. One-dimensional semiconductor nanomaterials such as conducting-polymer nanomaterials [25–27], organic-inorganic nanocomposites [28], metal [29–32], metal oxides [33], carbon nanotubes [34–41] and semiconductor quantum dots [42–45] are extremely attractive for designing high-density protein arrays. Because of their high surface-to-volume ratio, electro-catalytic activity as well as good biocompatibility and novel electron transport properties make them highly attractive materials for ultra-sensitive detection of biological macromolecules via bio-electronic devices. Some nano-scale materials exhibited remarkable electron transport properties, which are strongly depend on their nanocrystalline structure. Particularly, nanomaterials with different shapes and sizes have different electrical conductance [12,21–23]. The electron transport properties of the electrode can be monitor by the change in electrical conductance of the fabricated electrode. For example, in the case of perfect crystalline silver nanowires having four atoms per unit cell, generally three conductance channels are found [46]. One- or two-atom defects, either by addition or removal of one or two atomss may disrupt the number of such conductance channels and may cause variations in the conductance. It has been observed that small changes in the surface conditions of the nanowires can cause remarkable changes in their transport behavior. Moreover, it has been reported that the change in electrical conductivity of the bio-electrode is influenced by minor surface perturbations such as binding of bio-macromolecular species on a long conduction channel. 1D semiconductor electronic biosensors, in particular, have active surfaces that can easily be modified for immobilization of numerous biomolecules [45]. Additionally, the sizes of biological macromolecules, such as proteins and nucleic acids are comparable to nanoscale building blocks. Therefore, any interaction between such molecules should induce significant changes in the electrical properties of 1-D nanostructures.

One-dimensional nanostructures offer new capabilities not available in larger scale devices (for example, study of single molecule properties) [45,47,48]. However, this advantage may not apply to many non-oxide semiconductor nanomaterials because their surfaces are not stable in an air environment, which leads to formation of an insulating native oxide layers and may degrade device reliability and sensitivity. Due to the extreme smallness of these nanomaterials, it is possible to pack a large number of bio-macromolecule-functionalized nanomaterials onto a remarkably small footprint of an array device. All these properties of the nano-scale materials strongly depend on the synthesis procedures used to grow them. As a result of continuous progress in synthesizing and controlling materials on the submicron and nanometer scales, novel advanced functional materials brings new possibilities for electrochemical biosensor construction and for developing novel electrochemical bioassays. When scaled down to a nanoscale, most materials exhibit novel properties that cannot be extrapolated from their bulk behavior. The interdisciplinary boundary between materials science and biology has become a fertile ground for new scientific and technological development. For the fabrication of an efficient biosensor, the selection of substrate for dispersing the sensing material decides the sensor performance.

The morphology of the nanomaterials is another important factor to make them functional and operational for the design of efficient electrochemical biosensors. It has been noticed that the morphology of the nanoscale materials such as shape, size, diameter, surface condition, crystal structure and its quality, chemical composition, crystallographic orientation along the axis *etc.* are very important parameters, all of which influence the electron transport mechanism of nanomaterials [12,21,22,45]. These nanometer-scale electronic transducers reduce the pathway for direct electron communication between redox biomolecule to the electrode for sensitive and speedy detection of analyte without any hindrance. Therefore, extensive efforts have been made to synthesized novel morphological based nano-size materials such as nanowires, nanorods, nanotubes, nanofibers, nanobelts and nanorings, *etc.*, because these morphological nano-size materials based electrochemical biosensing devices show higher performance (sensitivity, selectivity, and real time detection limit) compared to those fabricated from other forms of the nanomaterials [21,33,45,47,48]. These novel nanomaterials with control of size, shape and structure can be tuned by altering the physical, chemical and biological routes.

Morphological based nanomaterials show new capabilities that are generated by combination of novel nanobuilding units and strategies for assembling them. These extraordinary electrocatalytic characteristics of the nanomaterials are being exploited in the fabrication of an efficient electrochemical biorecognotion device. In that respect, nanoscale materials have been used to achieve direct wiring of bio-macromolecules to electrode surface, to promote electrochemical reaction, to impose barcode for biomaterials and to amplify signal of biorecognition event. The resulting electrochemical nanobiosensors have been applied in the areas of cancer diagnostics and detection of infectious organisms [33]. Various kinds of nanomaterials, such as noble metal nanoparticles (Au, Pt), metal oxide nanoparticles, polymeric and inorganic-organic nanocomposites, carbon nanotubes (CNTs) and quantum dots (CdS, ZnSe, ZnS, CdSe and PbS), are being gradually applied to biosensors because of their unique physical, chemical, mechanical, magnetic and optical properties, and markedly enhance the sensitivity and specificity of detection. In this review, we illustrate the usefulness of nanoscale materials for the designing of efficient electrochemical DNA sensing device and also highlight the potential analytical applications in terms of nanostructured sensors and catalytic nanomotors.

A variety of synthesis processes have been proposed for the deposition of thin films on the conductive electrode surfaces for the fabrication of electrochemical bio-transducers. Among the synthesis processes for thin films on electrode surfaces, electrochemical deposition provides better results than others, because it permits control of the final properties of nanomaterials, such as morphology, size, thickness, length, diameter, orientation, and alignment on electrode surfaces (especially this last property fundamental to control the final analytic response). This is possible because all the electroanalytic parameters—such as the potential value, the current density, the deposition time, the electrical charge required for the growth, the supporting electrolyte and its ionic strength, the properties of the doping agents (due to the presence of specific functional groups, acting as stabilizing agent toward the polymeric films), and the pore membrane dimensions—can be controlled. These parameters played a crucial role in the design of sensitive electrochemical biosensing devices.

## 3. DNA Immobilization Techniques

Strategies of immobilization of ss-ODN probes onto a transducer surface to recognize specific diseases, including cancer, AIDS, bronchitis and bioterrorism agents play a fundamental role in rapid detection of genetic disorder. In addition to high reproducible sensitivity, specificity, operational stability, long-term use and detection of long linear concentration range of the analyte depend on the employed immobilization strategy on the electrode surface. The choice of the immobilization method depends mainly on the ss-ODN probe to be immobilized, the nature of the solid surface and the transducing mechanism. Therefore, a successful transducing surface required some important parameters including selection of electrode materials, biocompatibility, nontoxicity, absence of diffusion barriers, stability with changes in temperature, pH, ionic strength or macro-environment, sufficient sensitivity and selectivity for the analyte of interest as well as low cost and ease of mass production.

The immobilization of the sensing bioelement (probe), which specifically recognizes the analyte (target), onto a transducing surface, is the key-step in the construction of biosensing devices. There are many methods to immobilize the bio-macromolecules such as adsorption, physical entrapment in gels or membranes, cross-linking, covalent binding, entrapment, encapsulation and others as use of solid binding matrices. The immobilization matrix may function purely as a support or may also be involved with mediation of the signal transduction mechanism. The purpose of any immobilization method is to retain maximum activity of the biological component on the surface of the transducer. The selection of an appropriate immobilization method depend on the nature of the biological element, type of the transducer used, physico-chemical properties of the analyte and the operating conditions for the biosensor. Physical adsorption of the bio-component based on van der Waals attractive forces is the oldest and simplest immobilization method. Generally, the adsorption of bio-macromolecules directly onto naked surfaces of bulk materials may frequently result in their denaturation and loss of bioactivity. However, the adsorption of such bio-macromolecules onto the surfaces of nanosized materials can retain their bioactivity because of the biocompatibility of nanoparticles. Since most of the nanosized materials carry charges, they can electrostatically adsorb biomolecules with different charges [26,30,31].

Besides the common electrostatic interaction, some nanosized materials can also immobilize biomolecules by other interactions. For example, it is reported that gold nanoparticles can immobilize ssDNA through the covalent bonds formed between the gold atoms and the amine groups and cysteine residues of proteins [29–31]. DNA sensors can be made by immobilizing single stranded (ss) DNA probes on the nanoscale materials electrode using electroactive indicators to measure the hybridization between DNA probes and their complementary DNA strands. The detection of specific DNA sequence by electrochemical process commonly rely on the attachment of a single-stranded (ss)-ODN probe onto a transducer surface to recognize–via base pairing–its complimentary target sequence. Therefore, these materials should either possess the necessary functional groups on the surface needed for the attachment of ssDNA molecules that can be easily functionalized. Recent efforts have led to a host of new immobilization strategies for electrical detection of DNA hybridization. Such electrochemical avenues for generating the hybridization signal are the subject of the present review.

## 4. Electrochemical DNA Biosensors Based on Nanoscale Materials

Recent years have witnessed the advancement of powerful electrochemical DNA biosensors based on nano-sized labels and amplification platforms. Electrochemical DNA biosensors are of major interest due to their tremendous promise for obtaining sequence-specific information in a faster, simpler and cheaper manner, compared to the traditional techniques. Recent advances in developing such devices open new opportunities for DNA diagnostics. DNA biosensors, based on nucleic acid recognition processes are rapidly being developed towards the assay of rapid, simple and economical testing of genetic and infectious diseases. Electrochemical detection of DNA hybridization usually involves monitoring of a current response, resulting from the Watson–Crick base-pair recognition event into a readable analytical signal, under controlled potential conditions. A basic DNA biosensor is designed by the immobilization of a single stranded oligonucleotides probe on a transducer surface to recognize its complementary (target) DNA sequence via hybridization. The probe-coated electrode is commonly immersed into a solution of a target DNA whose nucleotide sequence is to be tested. When the target DNA contains a sequence which matches that of the immobilized ODN probe DNA, the hybrid duplex DNA which is formed at the electrode surface is known as the hybrid. Such hybridization event is commonly detected via the increased current signal of an electro-active indicator (that preferentially binds to the DNA duplex), in connection to the use of enzyme labels or redox labels, or from other hybridization-induced changes in electrochemical parameters (e.g., capacitance or conductivity).

Nanosized materials act as enhancing agents for effective acceleration of electron transfer between electrode and detection molecules, thus leading to more rapid current responses for target molecules. The advantage of the resulting transducers enhanced the quantity and activity of the immobilized redox active biomacromolecules (both useful to increase the sensitivity and stability of the resulting sensors). Considering their unique chemical physical properties, in particular the high surface nominal area, nanomaterials provide interesting opportunities for development of novel design of biosensors. In the following sections, we addressed the application of the nanomaterials involved in the construction of portable electrochemical DNA biosensing devices. As will be illustrated the success of such devices requires a proper combination of nanomaterials surface chemistries, DNA-recognition, and electrical detection protocols.

### 4.1. Use of Polymeric Nanoparticles for DNA Biosensors

The most widely investigated polymeric nanomaterials used for bio-macromolecule immobilization are conducting polymers including polyaniline, poly(phenylenevinylene), polypyrrole, polythiophene polyacetylene and polyindole [49]. The unique electronic structure of polymeric nanomaterials is responsible for their remarkable high electrical conductivity, ease of processibility, low ionization potentials, good environmental stability and high electron affinity [26]. Conductivity exhibits a strong dependence on solution pH and oxidation state. Conducting polymeric materials retain the exclusive properties of nanomaterials like as large surface area, size, and quantum effect, which further increase the merit of conducting polymers in designing and making novel biosensors [25–27]. In terms of biological applications, the thickness and shape of the polymeric film, which is most important factor to control the electrochemical characteristics of the transducers, can be easily controlled in the nanometer to micrometer range by the modification of the deposition method. These excellent properties of the polymeric nanomaterials provide better signal transduction, enhanced sensitivity, selectivity, durability, biocompatibility, direct electrochemical synthesis and flexibility for the immobilization of biomolecules, including DNA [26]. Versatility of these polymers are determined by the following: its biocompatibility; capability to transduce energy arising from interaction of analyte and analyte-recognizing-site into electrical signals that are easily monitored; capability to protect electrodes from interfering materials; easy ways for electrochemical deposition on the surface of any type of electrodes. Nowadays polymeric nanomaterials are becoming major tools for nanobiotechnological applications.

A thin film of polymeric nanomaterials having both high conductivity and fine structure on the nanoscale is a suitable substrate for immobilization of single strand-ODNs for electrochemical DNA hybridization detection. Nie *et al.* [50] presented a simple and label-free electrochemical sensor for detection of DNA hybridization based on a nanostructured conducting polymer, poly(indole-6-carboxylic acid). Covalently grafted 18-mer amino-substituted ODN probe onto the polymer surface displayed dynamic determination range for complementary target ODN from 3.5 × 10^−10^ mol L^−1^ to 2.0 × 10^−8^ mol L^−1^ and the corresponding detection limit was 5.79 pmol L^−1^ [50]. A sexually transmitted disease (*Neisseria gonorrhoeae*) biosensor was developed on electrochemically polymerized nanostructured polyaniline (nsPANI) film deposited onto indium-tin-oxide (ITO) electrode. The probe ssDNA was covalent attached to the functionalized nanostructured polyaniline surface through a cross-linking agent avidin–biotin coupling agent. The nsPANI amplify DNA recognition and transduction events, which is applied for ultrasensitive electrochemical detection of target DNA. Renu *et al.* observed improved detection limit of complementary target ODN up to 0.5×10^−15^ M within 60 s of hybridization time at 25 °C. The proposed approach is highly sensitive and selective for detection of specific nucleic acid and can be used to distinguish the presence of *N. gonorrhoeae* from *Neisseria meningitidis* and *Escherichia coli* culture and spiked samples from the urethral swabs of the patients. This biosensor was used for clinical samples [51]. Ghanbaria *et al*. [52] have applied electrochemically deposited nano-structured polypyrrole film onto Pt electrode for DNA sensing. Scanning electron microscopy (SEM), cyclic voltammetry (CV) and electrochemical impedance spectroscopy (EIS) were used to analyze the surface morphology and analytical characteristics of the electro polymerized polypyrrole film deposited on the Pt electrode. The proposed biosensor has good dynamic range, correlation coefficient (0.05–1.0 μM and 0.9983, respectively) and low detection limit (0.02 μM) [52].

In addition to nanowires, nanofibers, nanotubes and nanorods of polymeric materials see growing interest in the design and development of electrochemical transducers. The ease of fabrication and ability to manipulate their electrical, magnetic, and optical properties make them attractive for the construction of DNA biosensing devices. Nanotubes of conducting polymers make a channel for transferring the electron from the redox active site of ssDNA molecule to electrode surface. Chang *et al.* [53] have been growing highly organized conducting polyaniline nanotubes on a well-controlled nanoscale dimension on graphite electrodes using a magnetron sputtering method, followed by two-step anodization in oxalic acid at 40 V and 4 °C to create an alumina template of nanopore arrays. This process allows orientation and location control of the nanotubes, which are applied to immobilized 21-mer oligonucleotides (ODN) probes for the fabrication of electrochemical DNA biosensor. The analytical characteristics of the resulting biosensors were optimized by using differential pulse voltammetry (DPV). Conducting PANI nanotube arrays have signal enhancement capability, allowing the DNA biosensor to readily detect the target ODN at a concentration as low as 1.0 fM (∼300 zmol of target molecules). They found that the biosensor displays good capability of differentiating the perfect matched target ODN from one-nucleotide mismatched ODNs even at a concentration of 37.59 fM [53]. Due to high mechanical and chemical stability and good electrical conductivity polymeric materials amplify the electrochemical signal for sensitive detection of analyte. In this context a number of reports have been published in the literature [55–64].

The majority of approaches detecting hybridization events involve the covalent attachment of appropriate ODNs on conductive electrode substrates including inherently conducting polymers. For example, it has been shown that ODNs can be covalently attached to polyaniline monomers forming electrochemically conductive electroactive copolymers. Alternatively, ODNs directly covalently attached to polymeric surface after polymer synthesis. According to them, the conductance of the polymeric materials was changed upon interaction with the complementary DNA, enabling the sensing of the ODN [51–63]. The specific hybridization of grafted ODNs with the complementary nucleotide target induces a modification of the electrochemical behavior of the conductive polymer backbone. When non-complementary DNA (one base mismatch) was introduced in the sample, no response was observed, verifying the specificity of the sensor [64].

Additionally, in comparison to metal nanoparticles, conducting polymeric nanomaterials have some advantages including low-temperature synthesis, tunable conductivity, and no need for purification, endopening, or catalytic deposition processing. Unfortunately, polymeric nanomaterials are usually less favorable as the element in biosensor construction because of their relative low conductivity than the carbon nanotube as well as their nonoriented nanofiber morphology, leading to low detection sensitivity.

### 4.2. Use of Metal Nanoparticles for DNA Biosensors

In the emergence of nanotechnology noble metal nanoparticles are opening new horizons in the application of analytical chemistry. Due to their special sizes, noble metal nanomaterials display novel physical and chemical properties, such as the nanoscale effect and surface effect, *etc.* Catalysis effect is another outstanding characteristic of the transition metal nanomaterials, especially the noble metals, which extensively applied for many chemical synthesis reactions [28–31]. Similarly, metal nanoparticles are also both heterogeneous and homogeneous catalysts. The catalysis takes place on the active sites of the surface of metal nuclei (*i.e.*, the mechanism is similar to conventional heterogeneous catalysis). Owing to superior stability and complete recovery in biochemical redox processes, noble metal nanoparticles have been applied as catalysts in numerous biomedical applications [28–30]. Additionally, noble metal nanoparticles are redox active nanomaterials, open the possibility of the miniaturization of the sensing devices to the nanoscale, which offer excellent prospects for chemical and biological sensing. These noble metal nanoparticles are widely recognized as an ideal support for fabricating electrochemical biosensors.

Metallic nanoparticles not only improve the sensing properties of the biomolecules but also enhance the electron communication rate between redox active ssDNA species and electrode surfaces. However, nanoparticles have been used as labels in electrochemical DNA sensing to increase the loading of electroactive species for signal amplification. Many researchers have explored the properties of Au, Ag, Pt and Pd nanoparticles for the designing of amperomatric bioelectronic device [64–67]. Mirkin reported gold nanoparticle-based electrochemical DNA chips [64]. Wang and his coworkers [65,66] developed powerful stripping voltammetry electroanalytical technique based on metal nanoparticles for determination of trace amount of target DNA hybridization. This technique is highly sensitive and offers remarkably low detection limits (picomolar). Recent activity has led to highly sensitive nanoparticle-based stripping electrical bioassays applied for electrochemical DNA sensors with sensitivities in the pico- and femtomolar range [65,66]. Similar group in another approach have applied nanoparticle-based protocol for detecting DNA hybridization based on a magnetically induced solid-state electrochemical stripping detection of metal tags [67]. Zhu *et al*. [68] employed multi-walled carbon nanotubes (MWCNTs) and Pt nanoparticles dispersed in Nafion modified GCE for construction of sensitivity-enhancing electrochemical DNA biosensing ability. Nafion as a biopolymer has excellent film forming ability capable of higher loading of the ODN onto the bioelectrode for lower range determination of target DNA. The performance of the biosensor showed sensitive determination of DNA hybridization with a linear concentration from 2.25 × 10^−7^ to 2.25 × 10^−11^ mol L^−1^with detection limit 1.0 × 10^−11^ mol L^−1^. Qing *et al*. [69] electrodeposited Pt nanoparticles on GCE surface for electrochemical hybridization determination of specific deoxyribonucleic acid sequence in genetically modified soybean. A linear calibration graph was observed for the complementary DNA over a concentration range of 2.14 × 10^−9^−2.14 × 10^−7^ M and detection limit 1.0 × 10^−9^ M.

Chang *et al*. [70] applied palladium(Pd) nanoparticles combined with MWCNTs dispersed in Nafion modified on GCE that showed a much enhanced signal for the sensitive determination of target DNA hybridization. The DPV of the electrode before and after hybridization was determined in the presence of a methylene blue (MB) as an indicator at −0.32 V. Due to large surface area of Pd nanoparticles and MWCNTs, they accelerate the electron transfer rate of redox MB for selective and sensitive determination of DNA hybridization signal. The resulting electrochemical biosensor showed linearity for target DNA from 7.5 × 10^−13^ to 2.3 × 10^−9^ M and detection limit 1.2–10^−13^ M.

Gold nanoparticles are another important nanomaterial used for DNA hybridization detection. In this approach, thiol molecules are used to stabilize gold nanoparticles by covalent Au–S bonds. In addition, strong covalent bond between gold nanoparticles and −SH groups could offer an opportunity to construct multilayer films using cross-linkers with these functional groups. The chemical bonds formed between the Au nanoparticles and the enzymes facilitate the redox process and enhance the performance of the biosensor. At solid electrode surfaces, the electrochemical oxidation of DNA is associated with the irreversible oxidation of guanine and adenine residues, with a great enhancement of the analytical signal. A novel method for selective and sensitive recognition of complementary DNA by chemically grafting probe ssDNA onto functionalized gold nanoparticles was presented by Glynou *et al*. [71]. The gold nanoparticles amplify DNA recognition and transduction events, which may be used as an ultrasensitive method for electrical biosensing of DNA or proteins. Multilayered uniform self-assembled structures have been formed for co-adsorption of probe ssDNA-functionalized gold nanoparticles. These have been used as scaffolds for detection of hybridized DNA. Self assembled monolayer required very small amount of ODN for covalent attachment to the surface functional groups, resulting in the binding of desired molecule in the near vicinity of the electrode surface and act as a molecular wire between biomolecule and the electrode surface. The resulting biosensor showed an enhanced peak current due to the multilayered gold nanoparticles not only provide a biocompatible microenvironment for the protein to undergo direct electron transfer reactions but also amplify the electrochemical signal by increasing the binding sites for the protein immobilization. The proposed biosensor was linear in the concentration range from 2 × 10^−9^ to 1 × 10^−7^ M with a detection limit of 6.7 × 10^−10^ M [72].

Hu *et al*. [73] developed nanoporous gold electrode and multifunctional encoded Au nanoparticles for designing a sensitive electrochemical DNA sensor. The multifunctional encoded Au nanoparticles amplify the detection signal efficiently and could detect the DNA target quantitatively, in the range of 8.0 × 10^−17^−1.6 × 10^−12^ M and low detection limit upto 28 aM. Yang *et al*. [74] described electrochemical impedance measurements for detection of sequence-specific DNA, related to phosphinothricin acetyltransferase (PAT) trans gene in the transgenic plants, based on electro-polymerized poly-2,6-pyridinedicarboxylic acid film on GCE. A layer of Au nanoparticles was assembled on the fabricated electrode for covalent adsorption of probe ssDNA on the electrode surface. The hybridization events were monitored by CV and DPV measurements of the immobilized probe ssDNA using MB as indicator. The hybridization event led to a decrease of impedance values (R_et_) reflecting the reduction of the electrode resistance. The difference of *R*et value between the ssDNA/NG/PDC/GCE and hybridization DNA-modified electrode (dsDNA/NG/PDC/GCE) was used as the signal for detecting the PAT gene fragment with the dynamic range from 1.0 × 10^−10^ to 1.0 × 10^−5^mol/L with a detection limit of 2.4 × 10^−11^ mol/L [74]. The high efficiency of the biosensor arises from the combination of the electrocatalytic properties of Au nanoparticles with the biocompatibility and flexibility of the polymeric materials. The high sensitivity, selectivity and long lifetime of DNA sensors depends on the immobilization procedure of DNA probes onto electrode surfaces. Zhang *et al*. [75] described electrochemically entrapment of ssDNA molecules in a polymeric film followed by introduction of gold nanoparticles for DNA sensing. The polymeric materials enhanced the enzyme loading and stability of the bioelectrode and amplified the DNA hybridization signal efficiently, whereas gold nanoparticles promote the electron transfer reaction on the electrode surface for fast response time for the analyte detection.

A glassy electrode modified gold nanoparticles/cysteamine/polyglutamic acid was applied for immobilization of probe ssDNA linked covalently to the gold nanoparticles through 5′-thiol-linker. DPV technique was used for monitoring the DNA hybridization events. The same group found that, the reduction peak current was linearly increased with increasing the concentration of complementary target DNA from 9.0 × 10^−11^ to 4.8 × 10^−9^ M with a detection limit of 4.2 × 10^−11^ M [75]. In another approach carboxylic group functionalized MWCNTs were assembled onto electropolymerized aminobenzoic acid film on the surface of the GCE for the detection of target DNA 76]. The biosensor showed linear response within the concentration range of complementary ODN from 1.0 × 10^−12^ to 5.0 × 10^−9^ M with a detection limit of 3.5 × 10^−13^ M [76]. Another report from the same group described a DNA biosensor based on by layer-by-layer covalent attachment of gold nanoparticles (GNPs) and thiol group functionalized MWCNTs on an Au electrode [77]. The electrostatic layer-by-layer self-assembly onto CNTs carriers maximizes the ratio of DNA tags per binding event to offer the greatest amplification factor reported to date (showing that the probe DNA activity increases with the number of DNA layers). SEM, FTIR and CV were used for confirmation of the alteration in surface morphology after immobilization of probe ssDNA to the carboxylic group and formation of the product in the appropriate form. Due to the ability of CNTs to promote electron-transfer reactions, the high catalytic activity of gold nanoparticles and the sensitivity of presented electrochemical DNA biosensors are remarkably improved. In the same report the proposed DNA biosensor demonstrated excellent selectivity, reproducibility and stability in DNA hybridization assay. The detection limit of the method for target DNA was 6.2 pM and response current showed linearity in a wide concentration range of target DNA from 5.0 × 10^−10^ to 1.0 × 10^−11^ M [77]. In another approach, the same research group presented an amperometric DNA biosensor based on silver nanoparticles/poly(*trans*-3-(3-pyridyl) acrylic acid) (PPAA)/ with (MWCNTs–COOH)modified GCE [78]. The carboxyl group functionalized MWCNTs were deposited onto GCE using electro-polymerization followed by electrodepositing silver nanoparticles on the composite film for DNA detection. The hybridization events were monitored by DPV measurements of the intercalated adriamycin. This biosensor showed excellent electrochemical performance during DNA hybridization assays such as high sensitivity, reproducibility, stability and long linear concentration range from 9.0 × 10^−12^−9.0 × 10^−9^ M with a detection limit of 3.2 × 10^−12^ M [78]. Polyamidoamine and 3-mercapto-propionic acid modified Au electrode were used for immobilization of DNA on gold nanoparticles to obtain a stable recognition layer through biotin–avidin combination to detect complementary target, using signal amplification with Au nanoparticles and Ru(NH_3_)_6_]^3+^ as redox electro-active indicators [79]. The resulting biosensor showed a dynamic detection range of the sequence-specific DNA from 1.4 × 10^−11^ to 2.7 × 10^−14^ mol·L^−1^ and the detection limit 1.4 × 10^−14^ mol L^−1^. This DNA biosensor revealed low detection limit and excellent selectivity against two-base mismatched DNA [79].

Electrochemically deposited gold nanoparticles and then zirconia (ZrO_2_) film modified on GCE was used for electrochemical detection of DNA hybridization. MB was used as redox intercalator for identification of DNA hybridization. The sequence-specific detection of DNA hybridization of PAT gene in the transgenic plants was detected with a detection range from 1.0 × 10^−10^ to 1.0 × 10^−6^ mol/L, and detection limit of 3.1 × 10^−11^ mol/L [80]. Electrochemical performance of probe DNA assembled onto colloidal gold nanoparticles and carboxyl group-functionalized CdS nanoparticles-modified Au electrode was well preserved [81]. Due to the high surface energy of Au nanoparticles an increase in the electrode surface area for more binding amount of CdS finally enhanced the electrochemical responses. CdS nanoparticles were used for simple covalent linking of carboxyl acid groups to functionalized CdS with amino group of cysteine. The DNA immobilization and hybridization on the exterior of CdS nanoparticles was characterized with the use of Co(phen)_2_^2+^ as an electrochemical indicator. The biosensor quantified at a linear range from 2.0 × 10^−10^ to 1.0 × 10^−8^ M, with a detection limit of 2.0 × 10^−11^ M [81]. Ding *et al*. [82] utilized highly sensitive bioelectronic protocols for sequence specific detection of target DNA. Modified gold nanoparticles with CdS nanoparticles were applied to amplify the detection signal by an amidation reaction between bio-bar code binding DNA on the surface of Au NPs and mercapto acetic acid on the surface of CdS NPs. They optimized the electrochemical performance of the resulting biosensor in the concentration range of target DNA from 1.0 × 10^−14^ to 1.0 × 10^−13^ M. A detection limit of 4.2 × 10^−15^ M of target DNA was achieved [82].

Du *et al*. [83] reported a novel and sensitive sandwich electrochemical DNA biosensor based on the amplification of magnetic microbeads and Au nanoparticles modified with bio bar codes and PbS nanoparticles. This involves a sandwich bioassay based on magnetic microspheres were coated with four layers of polyelectrolytes in order to increase the number of carboxyl groups on the surface of the magnetic microbeads, which enhanced the amount of the captured DNA. They found that modified magnetic microbeads improved the sensing performance of the bioelectrode and amplified the electrochemical signal of DNA loading, sensitivity, selectivity and detection limit. The present DNA biosensor showed a linear relationship with the target DNA within the concentration range of 2.0 × 10^−14^ M to 1.0 × 10^−12^ M and a detection limit up to 5.0 × 10^−15^ M [83]. A novel strategy was proposed by Hu *et al*. [84] based on electrochemical stripping assay for ultrasensitive detection of target DNA hybridization. Semiconductor PbS nanoparticles was used as a tag for DNA hybridization detection and electrochemical stripping measurement of the lead ions. This group fabricated nanoporous gold electrode modified with single-stranded DNA (ssDNA). Au nanoparticles co-loaded with two kinds of ssDNA could detect target DNA upto a femtomolar concentration and exhibited excellent selectivity against one-base mismatched DNA and non-complementary DNA. The resulting DNA biosensor demonstrated a good linear relationship with the target DNA concentration in the range of 9.0 × 10^−16^ to 7.0 × 10^−14^ M with a detection limit of 2.6 × 10^−16^ M [84].

Electrochemical detection of short DNA ODN of the avian flu virus H5N1 with the sequence 5′-CCA AGC AAC AGA CTC AAA-3′ on a gold electrode surface was performed by Ting *et al.* [85] in connection with the use of silver nanoparticles as a label conjugated with a well-known DNA intercalator, doxorubicin. The observed Ag/AgCl redox process signal of the silver nanoparticle labels was subsequently used to quantify the amount of DNA. The proposed DNA biosensor achieved a detection limit upto1 pM [85]. Kong *et al*. [86] described an ultrasensitive electrical detection method of nucleic acids based on interdigited microelectrodes. They found attached hematin molecules with hybridized DNA to act as a catalyst to accelerate reduction of ammoniacal silver ions to form silver nanoparticles. Alteration in conductance of the silver nanoparticles directly correlated with the number of the hybridized DNA molecules. Under optimized conditions the biosensor was sensitive upto1 fM. The proposed biosensor was also applicable to the detection of RNA. Zhang *et al*. [87] have reported an attractive ultrasensitive electrochemical DNA biosensor based on highly characteristic solid state process. Functionalized silver nanoparticles with typical size 3–5 nm were used as an electroactive label on the surface of gold electrode modified with thiolated natural probe peptide nucleic acid (PNA) and 6-mercapto-1-hexanol as linker for detection of ODN from the H5N1 bird flu virus. The proposed biosensor has good response to DNA over a wide concentration range from 10 fM to 10 nM with a detection limit upto 10 fM. Silver nanocluster-modified gold electrode has been constructed and used for the detection of DNA hybridization. The resulting biosensor showed highly linear calibration plot over the entire DNA concentration range from 500–2,500 ng/mL [88].

### 4.3. Use of Metal Oxides Nanoparticles for DNA Biosensors

One-dimensional semiconductor metal oxide nanoparticles are a new class of advanced material used in the design and fabrication of electrochemical biosensors [32,47]. These materials improve the analytical capacities of sensor devices which are highly desired. The inorganic ceramics exhibit relatively high mechanical strength, enhanced thermal stability and negligible swelling in both aqueous and organic solutions compared to most conventional materials. Nanometer-scale metal oxides based electronic biosensors offer high sensitivity and real-time detection. For example, due to the high surface-to-volume ratio of the metal oxide nanoparticles, the detection sensitivity of the constructed transducers may be increased to a single-molecular detection level by monitoring the very small change in conductance caused by binding of biomolecular species on a long conduction channel. Nanostructured metal oxide electronic biosensors, in particular, have active surfaces that can easily be modified for immobilization of numerous biomolecules. However, this advantage may not apply to many non-oxide semiconductor nanomaterials because their surfaces are not stable in an air environment, which leads to formation of an insulating native oxide layer and may degrade device reliability and sensitivity. Metal oxide nanoparticles based electrodes solved this problem. Although, many metal oxide based electrodes have been fabricated for detection of DNA hybridization. Feng *et al.* utilized a CeO_2_/chitosan composite matrix for immobilization of probe single-stranded DNA (ssDNA) for construction of DNA biosensor related to the colorectal cancer gene. Chitosan introduced CeO_2_ nanocomposite matrix represented good biocompatibility, nontoxicity and excellent electronic conductivity, showing the enhanced loading of ssDNA probe on the surface of electrode. DPV was used to analyze the signal response of internal hybridization indicator MB and amount of colorectal cancer target DNA sequence. The proposed biosensor shows satisfactory reproducibility, selectivity and linearity in a wide concentration range from 1.59 × 10^−11^−1.16 × 10^−7^ mol L^−1^ with high detection sensitivity. The same investigators observed highest hybridization efficiency at 45 °C [89]. A novel nanocomposite membrane, comprising of nanosized shuttle-shaped cerium oxide (CeO_2_), single-walled carbon nanotubes (SWNTs) and room temperature ionic liquid (RTIL) 1-butyl3-methylimidazolium hexafluorophosphate, was developed on the glassy carbon electrode (GCE) for electrochemical sensing of the immobilization and hybridization of DNA. SEM micrographs were used for recognition of the nanosized shuttle-shaped cerium oxide and immobilization of DNA on the electrode surface. DPV and CV were employed to examine the surface properties and electrochemical characteristics of the constructed transducers. In the range of 1.0 × 10^−12^ mol/L to 1.0 × 10^−7^ mol/L, and detection limit 2.3 × 10^−13^ mol/L was checked for detection of sequence specific DNA of phosphoenolpyruvate carboxylase gene [90].

Zhu *et al*. [91] detected DNA hybridization on zirconia (ZrO_2_) thin film-modified gold electrodes as a sensing platform using the DPV technique. ODN probes were covalently attached via phosphate group at 5′end to the electrodynamically deposited zirconia thin films onto the bare gold electrode. Methylene blue was utilized as an electro-active labeling indicator to investigate the electrochemical DNA hybridization assay. The linearity of the biosensor was estimated under the target DNA concentration ranging from 2.25 × 10^−10^ to 2.25 × 10^−8^ mol L^−1^ with a detection limit of 1.0 × 10^−10^ mol·L^−1^ [91]. MWCNTs, ZrO_2_ nanoparticles doped chitosan-modified onto GCE was employed for immobilization of ODNs for sensitive detection of DNA hybridization using electroactive daunomycin as an indicator [92]. Chitosan was chosen as the material to form the membrane due to its excellent film-forming and adhesion abilities, together with its nontoxicity and biocompatibility. Moreover, chitosan contains amino groups, thus providing a hydrophilic environment, which is compatible with the biomolecules. SEM analysis confirmed the presence of MWCNTs and ZrO_2_. Coupling of MWCNTs with chitosan and ZrO_2_ nanoparticles provides enhanced electroactive surface area for higher amount loading of probe DNA and excellent electron transfer ability between the ODNs and the electrode surface. The response of the fabricated biosensor was linear under the logarithm target DNA concentration range from 1.49 × 10^−10^ to 9.32 × 10^−8^ mol·L^−1^ with a detection limit 7.5 × 10^−11^ mol·L^−1^[92].

Another strategy was proposed for the construction of DNA biosensor based on chitosan doped ZnO nanoparticles for voltammetric detection of DNA hybridization. The immobilization of the probe ssDNA is based on the absorption of the nanostructured ZnO [93]. The nanostructure ZnO greatly enhances the active surface available for ssDNA binding over the geometrical area. The resulting nanobiocomposite provides a shelter for the ODNs to retain its bioactivity under considerably extreme conditions and the ZnO nanoparticles in the biocomposite offer excellent affinity to probe DNA. The established biosensor was effective to discriminate the complementary target sequence and two-base-mismatched sequence, with a detection limit of 1.09 × 10^−11^ mol·L^−1^ of complementary target [93]. Unfortunately, owing to some drawbacks of doped nanomaterials films for construction of bioelectronic transducers, especially their thickness and brittleness, the practical applications of ceramic materials need to be improved by alteration in the fabrication methods. Efforts have been made to seek a new process which could overcome the disadvantages for biomolecule immobilization in biosensor construction. In recent years, some investigators have developed sol-gel derived matrices for immobilization of desired biomolecules to construct the electrochemical biosensors.

**Scheme 1.**
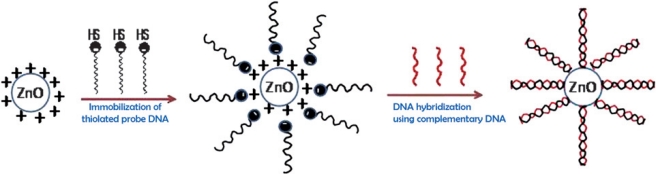
Sol–gel derived nanostructured ZnO-based STD sensor for the detection of *N. gonorrhoeae* [95].

Sol–gel materices can be prepared under ambient conditions and exhibit tunable porosity, high surface area, biocompatibility, optical transparency, excellent thermal stability, chemical inertness and negligible swelling in aqueous and non-aqueous solutions. Besides this, a sol–gel derived nano-porous film can retain its bioactivity in a given micro-environment and can be used for direct electron transfer between DNA active sites and the electrode. The high biomolecule loading per unit area and the optical transparency of the glass makes this approach particularly suitable for electrical signal transduction methodologies. Ansari *et al*. [95] exploited sol-gel derived nanostructured zinc oxide (ZnO) film deposited onto ITO glass substrate to immobilization of 20-mer thiolated ODN probe (th-ssDNA) for detection of target DNA (sexually transmitted disease - *Neisseria gonorrhoeae*) using a hybridization technique. X-ray diffraction, UV-Visible and SEM were applied to confirm the crystalline nature and morphology of the nanostructured ZnO film before and after probe ssDNA immobilization (Scheme 1). The response of the proposed biosensor was linear in the concentration range of target DNA from 0.000524 fmol–0.524 nmol, with a detection limit of 0.000704 fmol and hybridization time of 60 s [95].

A new approach has been developed by Zhu *et al.* based on Cu_2_O hollow microspheres consisting of Cu_2_O nanoparticles for the fabrication of an electrochemical DNA biosensor of hepatitis B virus. The lectrochemical performance of the biosensor showed sensitive determination of complementary target DNA sequences concentration ranging from 1 × 10^−10^ to 1 × 10^−6^ mol·L^−1^, with a detection limit of 1.0 × 10^−10^ mol·L^−1^. They found that the hollow Cu_2_O microspheres greatly enhanced the immobilization of the DNA probe on the electrode surface and improve the sensitivity of DNA biosensors [95]. A biosensor was fabricated by drop coating a carboxylic group functionalized magnetic nanobeads mixture onto the surface of GCE followed by the deposition of MWCNTs and 5′-NH_2_ capped probe sequence ODN by EDC solution based chemistry. High electron communication ability of the MWCNTs and magnetic enrichment improves the detection sensitivity of the proposed biosensor. In the range of 1.0 × 10^−13^−1.0 × 10^−6^ M, the concentration of the complementary sequence was linear with the response of the electrochemical signal of MB and the detection limit of target ODN was 43 fM [96].

Fe_2_O_3_ microspheres and self-doped polyaniline (PANI) nanofibers (copolymer of aniline and *m*-aminobenzenesulfonic acid) modified carbon ionic liquid electrode was used for immobilization of probe ssDNA for sensitive impedomatrically detection of sequence-specific DNA of phosphoenol-pyruvate carboxylase (PEPCase) gene [97]. Fe(CN)_6_]^3−/4−^ was employed as an internal indicator. Strong adsorption ability of Fe_2_O_3_ microspheres and excellent conductivity of self-doped PANI nanofibers (copolymer of aniline and *m*-aminobenzenesulfonic acid) enhanced the sensitivity of DNA hybridization recognition. In the same study, DNA hybridization events were monitored with a label-free EIS strategy. The response of the optimized biosensor was measured under the wide concentration range from 1.0 × 10^−13^ to 1.0 × 10^−7^ mol/L, with a detection limit 2.1 × 10^−14^ mol/L [97]. Shrestha *et al*. [98] have applied a new biosensing strategy based on modified rare earth semiconductor oxide followed by surface-immobilized single-stranded ODN for label free rapid detection of DNA hybridization by impedomatricaly, change in electrical impedance curve was used as a detection signal. They observed shifts in impedance curves because of changes in the interfacial electrical properties of the adsorbed single stranded nucleic acid and its complementary partner upon hybridization with the complementary oligonucelotide strand [98]. In a similar report thiol-modified ODN was immobilized on the surface of praseodymium oxide for impedomatric detection of unlabeled DNA hybridization [99]. Atomic force microscopy image were used to investigate the surface topographical features of the deposited film before and after immobilization of probe DNA. The proposed electrochemical AC impedomatric biosensor showed ultrasensitivity for the detection of complementary ODNs in solution without the use of label reagent.

### 4.4. Use of Inorganic-Organic Nanocomposites for DNA Biosensors

Since last decade, organic-inorganic hybrids nanocomposite materials have attracted substantial attention from many researchers because they combine the potential distinct properties of organic and inorganic components within a single molecular composite [11,27,32]. Organic materials offer structural flexibility, convenient processing, tunable electronic properties, photoconductivity, efficient luminescence and the potential for semiconducting and even metallic behavior. Inorganic compounds provide the potential for high electron carrier mobilities, band gap tunability, a range of magnetic and dielectric properties, and thermal and mechanical stability [100,101]. In addition to combining distinct characteristics, new or enhanced phenomena can also arise as a result of the interface between the organic and inorganic components. These hybrid nanocomposites materials provide enhanced dual characteristics which efficiently retain the bioactivity of immobilized probe ssDNA for construction of biorecognition transducers. An organic–inorganic nanocomposite membrane is quite promising and has been utilized as a sensing platform in electrochemical DNA biosensor. Some reports on hybride nanocomposites have been discussed in previous sections.

### 4.5. Use of Quantum Dots for DNA Biosensors

Semiconductor nanomaterials (CdS, ZnS, PbS, GaN) were used for designing an amperometric DNA biosensor. Owing to their unique (size-tunable fluorescent) properties, the intrinsic redox properties and the sensitive electrochemical stripping analysis of the metal components of semiconductor nanoparticles cause the labels in the electrochemical biosensor to be very sensitive. The concept was first demonstrated by Wang’s group using semiconductor nanoparticle labels for the electrochemical DNA hybridization assay. Semiconductor nanoparticles maintained the bioactivity and the structure of probe ssDNA molecules and also electrocatalyzed the reduction of dissolved oxygen, resulting in a significant increase of the reduction peak current. In recent years, several inventive designs for electrochemical DNA biosensors based on semiconductor quantum dots have appeared. Wang *et al*. [102–105] reported on electrochemical assays based on quantum dot nanocrystals as tracers. These quantum dots exhibit sharp and well resolved stripping voltammetry signals proportional to the concentration of corresponding DNA targets due to the well defined oxidation potentials of the metal components. The calibration plots were linear for the resulting biosensor with the lowering detection limit 2.7 pM, correlation coefficients, 0.979 (T1) and 0.975 (T2) [105]. In a similar strategy Hansen *et al.* [43] utilized CdS nanoparticles for label free electrochemical sensing of the target DNA. The proposed stripping voltammetry method offer excellent sensitivity up to 0.1 fmol of target DNA. Ding *et al.* [42] reported a sandwich electrochemical immunoassay protocol for quantitative detection of target DNA or other proteins based on the use of different semiconductor nanoparticle tracers (CdS, ZnS, and PbS). The fabricated sandwich electrochemical biosensor offer reliable low detection limit of 9.6 pg/mL [42]. Chen *et al*. [42] have developed GaN nanowires for label free electrochemical detection of target DNA (anthrax lethal factor sequence) using dual route - EIS and photoluminescence (PL) -measurements. The resulting GaN nanowires biotransducer showed enhanced sensitivity to surface-immobilized DNA molecules as nanowires provided high surface binding energies for more binding sites to probe DNA and surface-enhanced charge transfer capability to the analyte. This novel biosensor revealed excellent selectivity and specificity, down to picomolar concentration, high response sensitivity and a low detection limit useful for potential applications [42].

### 4.6. Use of CNTs for DNA Biosensors

Carbon nanotubes (CNTs) are promising carbonaceous materials which have attracted considerable attention by many researchers because of their unique structure-dependent electrical, chemical and mechanical properties. The unusual properties of the CNTs owing to the covalent sp^2^ bonds and tubular structure with large length/diameter ratios render them excellent candidates for biosensor or bioreactor applications. CNTs can be divided into single-wall carbon-nanotubes (SWCNT) and multi-wall carbon-nanotubes (MWCNT). SWCNT possess a cylindrical nanostructure (with a high aspect ratio), formed by rolling up a single graphite sheet into a tube. SWCNT can thus be viewed as molecular wires with every atom on the surface. CNTs are used in composite materials in electronic devices, as sensors, actuators, field emitters, energy storage media and biomaterials. The high stability of the CNTs in an oxidative environment makes them not only excellent catalyst support materials but also high-performance catalysts for hydrocarbon oxidation. In addition, processing in oxidative environment is one of the most widely used methods for purificating or reshaping the original structure of carbon-based materials or for tailoring their physical, chemical, and electronic properties by introducing oxygenated groups in the C cage. The oxygen functional groups can convert the metallic CNTs into semiconducting, improve the adhesive properties, or selectively functionalize the surface to meet the application demands. Furthermore, the use of CNTs as analytical tools, and the construction of nanodevices and nanosensors based on CNTs are other exciting areas of development for modern analytical science. The general roles of CNTs in analytical chemistry were recently reviewed [107–117].

Considering, in particular, the role of CNTs in electroanalytical chemistry, properties such as good biocompatibility, huge high surface area, wide electrical windows, flexible surface chemistry, ease to functionalization for biomolecule co-adsorption, enhanced electronic conductivity and a high mechanical resistance have driven an impressive research effort in electroanalytical applications. Recently, the important feature and possible potential applications of CNTs were extensively reviewed [107–124]. The electrode fabrication techniques using CNTs and the hybridization indication techniques both play important roles in developing ultrasensitive, selective and miniaturized electrochemical DNA biosensor for quick and reliable DNA sequence analysis in practical application, such as early cancer detection and point-to-care use. Due to their huge surface energy and flexible surface chemistry to functionalize biomolecules, CNTs accelerate electron-transfer rate between the redox active ssDNA molecule and electrode. CNTs increase the attached DNA amount on the CNTs-based substrate surface, it also can concentrate a great number of enzyme or electroactive nanoparticles to indicate DNA hybridization. Owing to their excellent electro-transfer properties CNTs can amplify the electrochemical signal of DNA hybridization. All of these amplification factors have offered promising prospects for fabricating highly sensitive electrochemically DNA biosensing protocols. A biosensor based on chitosan doped with CNTs was successfully used to detect salmon sperm DNA [117–124]. Chitosan doped CNTs matrix deposited onto graphite electrode co-immobilized fish sperm DNA for detection of salmon sperm DNA. Chitosan is a biopolymer with highly stable mechanical and chemical properties and have a strong adhesive nature towards the substrate. Chitosan was widely used as an effective dispersant of CNTs. It provides large surface area for the covalent immobilization of ODNs, and therefore it enhances higher DNA loading and longer detection range of the analyte. MB was employed as a redox active indicator for electrochemically quantitative detection of DNA hybridization signal. It was found that CNTs can enhance the electroactive surface area threefold (0.28 + 0.03 and 0.093 + 0.06 cm^2^ for chitosan–CNT- and chitosan-modified electrodes, respectively) and can accelerate the rate of electron transfer between the redox-active MB and the electrode. A low detection limit of 0.252 nM fish sperm DNA was achieved, and no interference was found in the presence of human serum albumin. The DPV signal of MB was linear over the fish sperm DNA concentration range of 0.5–20 nM [125].

An interesting approach involves assembling of a DNA electrochemical biosensor based on chitosan doped MWCNTs deposited onto SPCE [126]. Analytical performance of the bionanocomposite transducer was investigated using DPV technique with the DNA redox marker [Co(phen)_3_]^3+^, CV and EIS with [Fe(CN)_6_]^3−^ as a redox probe in a phosphate buffer solution (PBS), respectively. Comparative studies between DNA/MWNT-CHIT/SPCE and DNA/CHIT/SPCE matrices have been proposed to confirm the deep DNA damage by using CV and electrochemical impedance spectroscopy techniques [126]. The remarkable electrical properties of CNT suggest the possibility of developing superior electrochemical sensing devices, ranging from amperometric enzyme electrodes to label-free DNA hybridization biosensors. The tailored electronic conductivity of CNTs, coupled with their ease of processing/modification and rich chemistry, make them extremely attractive as 1-D sensing materials. Hembram *et al*. [127] studied the electrical and optical properties of MWCNTs/DNA nanocomposite. CNTs were covalently bonded to DNA at the ends of defect sites and the wrapping of DNA on the CNTs is due to van der Waals force. They also found enhanced conductivity of the CNTs nanocomposite with increased DNA concentration [127]. CNTs facilitate the electrochemical oxidation of DNA guanine nucleotide, which allows direct detection of DNA on the modified electrodes. The chemical composition of DNA can alter the electrochemical properties of nucleic acids containing DNA. Furthermore, the denaturation of native DNA improves the adsorption of biopolymer on CNTs and results in an increase in DNA oxidation current on the modified electrode. The resulting CNT-modified bio-electrodes demonstrate the feasibility of direct detection and characterization of DNA and DNA damaging factors [128]. A self-assembled MWNTs layer was developed on a gold substrate to covalently immobilize probe ssDNA. The DPV technique was applied to examine the alteration in hybridization between the probe and target DNA with the help of MB as an internal indicator. Same group compared the biosensing results obtained from self-assembled MWNTs and random MWNTs. Self-assembled MWNTs-based biosensor were found to have higher hybridization efficiency, high selectivity and long range hybridization detection limit [129]. In a similar report, carboxylic SWCNTs were self-assembled on an amine-modified platinum electrode surface and followed by the assembly of NH_2_-DNA with the carboxyl-amine coupling for co-adsorption of DNA oligoneucleotides [130]. Field Emission Electron Microscopy (FEG-SEM) images demonstrated the covalent immobilization of the probe DNA on the fabricated electrode surface. CV and UV–Vis spectroscopy were used to investigate the molecular interaction between DNA probe and riboflavin (VB2). The resulting biosensor exhibited high sensitivity and low detection limit for the tested riboflavin [130].

CNTs were used to obtain a fine dispersion in selected solutions or matrices. Several methods have been developed, including covalent or non-covalent modifications. Depending on the methods used, functional groups can be introduced onto the surface of nanotubes. This would endow CNTs with multifunctional applications by integrating other functional groups or materials onto their surfaces. A functionalized nanotube might have mechanical, optical or electrical properties that are different from those of the original nanotube. Therefore, it is an interesting area to functionalize CNTs for all kinds of applications. Gong *et al*. [131] prepared DNA–thionine–carbon nanotube (DNA–Th–CNT) nanocomposites for immobilization of DNA on the surface of CNTs via thionine (Th). The fabrication process of nanocomposite was characterized using Raman spectroscopy, UV–vis spectroscopy, AFM and SEM. Thionine has excellent electron facilitating properties and efficiently accelerate the electron communication rate between the redox active species and electrode surface. In addition, thionine can retain the native secondary conformational structure of DNA molecules after their immobilization onto the bioelectrode. The functionalized CNTs have good quality electrochemical responses with long-term stability for potential use in the DNA biosensor field [131].

Tam *et al*. [132] have studied a covalently immobilized probe DNA on MWCNTs for direct and label-free detection of influenza virus (type A). The investigators used FTIR and Raman spectra for the confirmation of covalent bonding in between amine and phosphate groups of the DNA sequence. The fabricated DNA biosensor can detect target DNA up to 0.5 nM and the response time of DNA sensor is approximately 4 min. Moreover, they measured the electrical conductivity of the modified bioelectrode as a response signal of the biosensor, which was altered by DNA hybridization [132]. Thus, a novel strategy of altering the electronic properties of nanotubes are done either by chemically functionalizing them with a moiety or by altering the structure whose intrinsic properties are electrically configurable.

Zhu and co-workers [133] presented a very attractive work about non-covalent functionalization of MWCNTs sidewalls for immobilization of poly(amidoamine) dendrimer to be used for the fabrication of efficient electronic transducers to form the DNA biosensors. They found that G2-PAMAM dendrimer attached with MWNTs electronic transducer having a large number of amino groups on the surface increase the covalent bonding of probe DNA. This results in increase in the sensitivity and selectivity of the impedimetric biosensor for the target DNA with a low detection limit down to 0.1 pM. The Fe(CN)_6_]^3−^ was used as an electroactive indicator for DNA hybridization detection. The interfacial charge-transfer resistance of the bioelectrode was altered as the concentration of the target DNA was changed as indicated by the response signal of the transducer. The constructed biosensor exhibited linearity of the target DNA within a concentration range from 0.5 to 500 pM with a detection limit of 0.1 pM (*S*/*N* = 3). The new proposed method is simple, sensitive and reliable and could be reasonably useful for practical applications [133].

In another application, MWCNTs dispersed in dimethylformamide or aqueous sodium dodecyl sulfate (SDS) solution mixed into colloidal gold nanoparticles in phosphate buffered saline (PBS) were deposited on silver paste carbon electrode (SPCE) used as the signal transducer of a dsDNA-based biosensor. MWNTs in SDS solution based transducer revealed substantial enhancement in the electrochemical response. The fabricated biosensor was tested on berberine and isoquinoline plant alkaloid. They evaluated the anticancer effect of berberine on target DNA. The effect was found to be berberine concentration dependent in the range 75 to 50 μg·mL^−1^ [134]. Carboxyl group functionalized MWCNTs modified on electropolymerized aminobenzoic acid, covering the surface of the GCE, were applied for fabrication of sensitive electrochemical DNA biosensors for the detection of target DNA hybridization [77]. SEM, CV and EIS were used to investigate the electrode surface texture and electrochemical characteristics before and after enzyme immobilization. For covalent immobilization of DNA molecules Au nanoparticles layer was introduced onto the nanocomposite electrode surface. Gold nanoparticles promote the electron transfer rate between the redox active DNA species and the electrode surface. Under optimized conditions, DNA hybridization current was monitored by a DPV technique. The biosensor had linearity in a wide concentration range of the complementary ODNs from 1.0 × 10^−12^ to 5.0 × 10^−9^ M with a detection limit of 3.5 × 10^−13^ M [77].

Ye and Ju [135] reported the use of a screen printed carbon electrode modified with MWCNTs for the fast and sensitive detection of DNA and RNA from the electrooxidation of guanine and adenine residues catalyzed by MWCNTs. The proposed transducer could detect calf thymus ssDNA concentration ranging from 17.0 to 345 μg·mL^−1^ with a detection limit of 2.0 μg·mL^−1^ at 3*σ* and yeast tRNA ranging from 8.2 μg·mL^−1^ to 4.1 mg·mL^−1^ [135]. Wang *et al*. [136] employed CNT-modified GCE electrochemical transducers. They examined the attractive performance of the enzyme based electrochemical biassaays of DNA hybridization. CNT based electrochemical transducers were used for ultrasensitive electrical bioassays of proteins and DNA. The unique electronic, chemical, and mechanical properties of CNTs make them extremely attractive for electrochemical sensors. Most CNT-sensing work has focused on the ability of surface-confined CNTs to promote electron-transfer reactions involved in biocatalytic devices [138,139]. In another approach, CNT amplification platform combined with CdS particles have been reported by Wang *et al*. [139]. CNTs were utilized as supporting materials to concentrate nanoparticles or enzyme molecules on their surfaces as a new and more powerful DNA hybridization indicator than using only a single nanoparticle or enzyme molecule. Due to the large surface area of the CNTs, a larger number of octadecanethiol-capped CdS nanoparticles can be attached onto acetone-activated CNTs under hydrophobic force. The whole complex is then used as a hybridization indicator to be labeled at probe 2 DNA. After hybridization in a sandwich manner (probe 1-target-probe 2), these CdS nanoparticles are dissolved into Cd^2+^ for stripping voltammetry detection. Because 500 CdS particles can load on an individual CNT, the detection limit is consequently improved to 500-fold as compared with single CdS nanoparticle labeling technique.

## 5. Conclusions and Future Prospects

Nanostructured materials are opening new horizons in the development of electrochemical DNA biosensing devices. Such DNA biosensing devices could be useful for diagnosing and monitoring infectious diseases, monitoring the pharmokinetics of drugs, detecting cancer and disease biomarkers, analyzing breath, urine and blood for drugs of abuse, detecting biological and chemical warfare agents, and monitoring pathogens in food, among other conceivable applications. The unique and attractive properties of nanostructured materials present new opportunities for the design of highly sophisticated electroanalytical DNA biosensing devices. Due to their high surface area, nontoxicity, biocompatibility and charge-sensitive conductance of nanomareials they act as effective transducers in nanoscale biosensing and bioelectronic devices. These nanostructured materials based electrochemical DNA devices have a number of key features, including high sensitivity, exquisite selectivity, fast response time and rapid recovery (reversibility), and potential for integration of addressable arrays on a massive scale, which sets them apart from other sensors technologies available today. The sensitivity of the sensor depends on the dimensions and morphological shape of the nanomaterials involved. Therefore, some morphological (nanotube, nanowires, nanofibers, nanorods) based biosensing transducers could function as effective mediators and facilitate the electron transfer between the active site of probe DNA and surface of the electrodes. The resulting nanostructures could be substantially stronger and lighter than conventional nanomaterials which are currently used in the construction of biosensing devices. There is an urgent need to develop an efficient and reversible effective electrochemical DNA biosensing device, capable of detecting analytes (target DNA) in small concentrations. In the near future, we argue that these advances could and should be developed at molecule level detection in simple nanosensor devices. To fully realize the potential applicability of nanostructures in electrochemical sensors, several issues related to their fabrication methods need to be addressed.

## Appendix

**Apendix 1.** Characterization parameters of the published literature reports

Immobilization Matrix	Linearity (mM)	Sensitivity	Detection limit (M)	Shelf life	Response	Ref.
Poly(indol-6-carboxylic acid)	3.5 × 10^−10^−2.0 × 10^−8^ mol/L	------	5.79 pmol/L	2 days	.......	49
Polyaniline/ITO	1 × 10^−16^–2.0 × 10^−6^ M	------	0.5 × 10^−15^ M	.....	60 s	50
Polypyrrole nanofibers	0.05–1.0 M	------	0.02 M	30 days	.......	51
Polyaniline nanotubes	3.759–755.7 fM	------	1.0 fM (∼300 Zmol)	------	------	52
Poly(thiophene-3-yl-acetic acid 1,3-dioxo-1,3-dihydro-isoindol-2-yl) ester	20–1,000 nmol	0.62 A/nmol	1 nmol	------	------	53
polypyrrole	0.1–0.5 M	------	1.6 fmol	2 month	------	54
polypyrrole	-----------	--------	100 pM (3 fmol)	------	------	55
Pt/CNTs	2.25 × 10^−7^–2.25 × 10^−11^ mol/L	------	1.0 × 10^−11^ mol/L	------	------	68
Pt nanoparticles	2.14 × 10^−9^−2.14 × 10^−7^ M	------	1.0 × 10^−9^ M	--------	--------	69
Pd nanoparticle/CNTs	3.5 × 10^−10^−2.0 × 10^−8^ mol/L	------	1.2 × 10^−13^ M	------	------	70
Au nanoparticles	0.36–2.8 pmol	--------	2 fmol	------	------	71
Au nanoparticles	2.0 × 10^−9^−1.0 × 10^−7^ M	------	6.7 × 10^−10^ M	------	------	72
Nanoporous Au electrode	8.0 × 10^−17^–1.6 × 10^−12^ M	------	28 aM	1 week	--------	73
NanoAu/Poly-2,6-pyridine-dicarboxylic acid	1.0 × 10^−10^–1.0 × 10^−5^ mol/L	------	2.4 × 10^−11^ mol/L	------	------	74
Au nano/cystamine/Poly(glutamic acid)	9.0 × 10^−11^–4.8 × 10^−9^ M	--------	4.2 × 10^−11^ M	--------	------	75
Au nano/Poly(p-aminobenzoic acid)/CNTs	1.0 × 10^−12^–5.0 × 10^−9^ M	------	3.5 × 10^−13^ M	------	------	76
Au nano/MWCNTs	5.0 × 10^−10^–1.0 × 10^−118^ M	------	6.2 pM	3 weeks	--------	77
Ag nano/poly3-(3-pyridyl)acrylic acid]/CNTs	9.0 × 10^−12^–9.0 × 10^−9^ M	------	3.2 × 10^−12^ M	2 weeks	------	78
Au nanoparticles	1.4 × 10^−11^–2.7 × 10^−14^ mol/L	--------	1.4 × 10^−14^ mol/L	------	------	79
Nano Au/zirconia	1.0 × 10^−10^–1.0 × 10^−6^ mol/L	------	3.1 × 10^−11^ mol/L	--------	------	80
Au nanoparticle/CdS nanoparticles	2.0 × 10^−10^–1.0 × 10^−8^ M	------	2.0 × 10^−11^ M	1 week	--------	81
Au /CdS nanoparticles	1.0 × 10^−14^–1.0 × 10^−13^ M	------	4.2 × 10^−15^ M	..........	------	82
Nano Au /PbS nanoparticles	2.0 × 10^−14^–1.0 × 10^−12^ M	------	5.0 × 10^−15^ M	8 hrs	------	83
Nanoporous Au/PbS nanoparticles	9.0 × 10^−16^–7.0 × 10^−14^ M	--------	2.6 × 10^−16^ M	------	--------	84
Silver nanoparticles	1 pM–10 nM	------	1 pM	--------	------	85
Ag nanoparticles	1.0 × 10^−11^–1.0 × 10^−15^ M	------	1.0 × 10^−12^ M	------	------	86
Ag nanoparticles	10 fM–10 nM	--------	10 fM	--------	--------	87
Silver clusters	500–2,500 ng/mL	------	100 ng/mL	------	------	88
CeO_2_/Chitosan	1.59 × 10^−11^–1.16 × 10^−7^ mol/L	------	1.0 × 10^−11^ mol/L	------	------	89
CeO_2_ nanoshttles/CNTs	1.0 × 10^−12^–1.0 × 10^−7^mol/L	------	2.3 × 10^−13^ mol/L	------	------	90
ZrO_2_/Au electrode	2.25 × 10^−10^–2.25 × 10^−8^ mol/L	--------	1.0 × 10^−10^ mol/L	--------	--------	91
CNTs/nano zirconia/chitosan	1.49 × 10^−10^–9.32 × 10^−8^ mol/L	------	7.5 × 10^−11^ mol/L	------	------	92
NanoZnO/chitosan	2.0 × 10^−6^–1.5 × 10^−5^ mol/L	------	1.09 × 10^−11^ mol/L	------	------	93
Sol-gel nanostructured ZnO	0.000524 fmol–0.524 nmol	------	0.000704 fmol	------	60s	94
Cu_2_O hollow microspheres	1.0 × 10^−10^–1.0 × 10^−6^mol/L	--------	1.0 × 10^−10^	--------	------	95
Magnetite nanoparticles	1.0 × 10^−13^–1.0 × 10^−6^M	------	43 fM	------	--------	96
Fe_2_O_3_/PANI/CILE	1.0×10^−13^–1.0 × 10^−7^mol/L	------	2.1 × 10^−14^ mol/L	------	------	97
Pr_6_O_11_/ITO	100–300 L	------	300 L	------	------	98
Chitosan/CNTs	0.5–20 nM	--------	0.252 nM	--------	------	124
Self assembled CNTs	5 × 10^−6^–3.0 × 10^−5^ mM	------	2.3 × 10^−4^ mM	------	--------	129
MWCNTs	1.0–10.0nM	0.06 mV/nM	0.5 nM	------	4 minutes	131
MWCNTs/DMF/SDS/GND	75–50 g/mL	------	......	------	------	134
MWCNTs/SPE	17.0–345 g/mL (CT-DNA) 8.2–4.1 g/mL (yeast tRNA)	--------	2.0 g/mL 1.0 g/mL	--------	--------	135
CNTs/GCE	20–120 μg^−1^	194.23 mglμ g^−1^	2.0 pg	------	------	136
